# Heterofullerene MC_59_ (M = B, Si, Al) as Potential Carriers for Hydroxyurea Drug Delivery

**DOI:** 10.3390/nano11010115

**Published:** 2021-01-07

**Authors:** Peng Wang, Ge Yan, Xiaodong Zhu, Yingying Du, Da Chen, Jinjuan Zhang

**Affiliations:** 1 College of Electronic and Information Engineering, Shandong University of Science and Technology, Qingdao 266590, China; phywangp@sdust.edu.cn (P.W.); ge_y0556@163.com (G.Y.); zhongxie152@163.com (X.Z.); phydyy2019@sdust.edu.cn (Y.D.); chenda@sdust.edu.cn (D.C.); 2 Department of Physics, Zhejiang University, Hangzhou 310027, China

**Keywords:** fullerene, DFT calculations, drug delivery, hydroxyurea

## Abstract

As a representative nanomaterial, C_60_ and its derivatives have drawn much attention in the field of drug delivery over the past years, due to their unique geometric and electronic structures. Herein, the interactions of hydroxyurea (HU) drug with the pristine C_60_ and heterofullerene MC_59_ (M = B, Si, Al) were investigated using the density functional theory calculations. The geometric and electronic properties in terms of adsorption configuration, adsorption energy, Hirshfeld charge, frontier molecular orbitals, and charge density difference are calculated. In contrast to pristine C_60_, it is found that HU molecule is chemisorbed on the BC_59_, SiC_59_, and AlC_59_ molecules with moderate adsorption energy and apparent charge transfer. Therefore, heterofullerene BC_59_, SiC_59_, and AlC_59_ are expected to be promising carriers for hydroxyurea drug delivery.

## 1. Introduction

Nanomaterials, such as C_60_ and its derivatives, have become increasingly important in gas sensors and biomedicine, especially in the field of drug delivery [1,2,3,4,5,6,7,8]. As the routine methods of drug administration, which suffered from the problems of lacking target specificity and unavoidable side effects, are in great need of improvement [9], much efforts have been done to develop new drug delivery systems based on nanomaterials. Nowadays, drug carriers based on nanomaterials can be used to control the release of a drug into systemic circulation, which could be achieved by triggered release at the target site by stimulus, such as changes in pH, application of heat, and activation by light [9].

In recent years, fullerenes have been regarded as one of the most exciting materials for developing new drug delivery systems, due to their unique properties including small dimensions, high surface-to-volume ratios, ease of functionalization, and biocompatibility [10,11,12]. For example, Wang et al. have successfully designed a nanoplatform based on a nucleus-like fullerene core and realized the precisely controlled delivery of theranostic agents both in vitro and in vivo [13]. Shi et al. found that PEI-derivatized fullerene could be used as drug delivery vehicles to achieve suppression of tumor growth without toxic effects on normal tissues [14].

It has been previously shown that doping with the impurity atoms could increase fullerene’s reactivity to different chemicals [15,16,17,18,19,20,21,22,23] and further improve the drug delivery properties. On the theoretical side, the interactions of heterofullerene (particularly the case of replacing a carbon atom with an impurity atom) with different drugs have been studied, including amantadine [19], 5-fluorouracil [20], amphetamine [21], phenylpropanolamine [22], and 4-Phenylpyridine [23]. On the experimental side, in 1996, Muhr et al. [24] reported the macroscopic preparation of C_59_B and C_69_B by arc evaporation of doped graphite rods in a modified fullerene reactor. So far, the synthesis of heterofullerenes, such as C_59_N [25], C_59_Si [26,27], C_59_O [28], C_59_P [29], C_59_As [30], C_59_Ge [30], and metal-doped C_59_M (M=Fe, Co, Ni, Rh, Pt, Ir) [31,32], have been performed successfully.

Hydroxyurea (HU), also known as Hydroxycarbamide, is an effective anti-cancer drug widely used in treating head and neck cancer, breast cancer, chronic myelocytic leukemia, and so on [33,34]. However, studies have shown that HU drug can cause some side effects, such as rashes, leukopenia, and leg ulcers during treatment [35,36], which significantly limits the application of HU in the treatment of cancer. Therefore, in order to reduce side effects and enhance the effectiveness, it is necessary to find a suitable drug delivery vehicle for HU drug [37,38]. Considering that the experimental procedure is expensive and time-consuming, we performed theoretical calculations with the aim to find potential carriers for HU drug delivery, which could offer more options to the experimental work.

In this work, we employ density functional theory calculations to accurately describe the adsorption of HU drug on the pristine C_60_ and heterofullerene MC_59_ (M = B, Si, Al), in order to reveal some clues for a drug delivery vehicle. In the following section, we present the details of the computational methods, which are then followed by the results and discussion. Finally, the general overview and conclusions are given.

## 2. Computational Details

In this work, all the geometry optimization and energy calculations were performed using the spin-polarized density functional theory (DFT) implemented in the DMol^3^ program package [39,40]. The gradient-corrected (GGA) exchange-correlation functional formulated by Perdue, Burke, and Ernzerhof (PBE) [41] along with the double numerical basis sets including d-polarization functions (i.e., the DNP set) are chosen here. In order to better describe the weak interaction between fullerene and drug molecules, we introduce a long-range dispersion interaction correction term produced by Grimme during the calculation [42]. In the self-consistent field calculations, the convergence criterion was set to 10^−6^ a.u. for energy and electron density. The geometries are fully optimized with no restrictions. In geometrical optimizations, we set the convergence criterion of 10^−3^ a.u. on the gradient and displacement and 10^−5^ a.u. on the total energy. To make sure the resultant structures belong to the real local minima, the normal-mode vibrational analysis was applied. No imaginary frequency was observed through vibration frequency analysis. The charge transfer from the HU molecule to fullerene is analyzed based on Hirshfeld charge analysis [43], which is based directly on the electron density as a function of space.

The adsorption energy (*E*_ads_) was defined as follows:

(1) $$E_{\mathrm{ads}}=E_{\mathrm{complex}}-E_{\mathrm{HU}}-E_{\mathrm{fullerene}\;\mathrm{or}\;\mathrm{heterofullerene}}$$

where *E*_complex_, *E*_HU_, and *E*_fullerene or heterofullerene_ are the total energy of the HU adsorbed on C_60_ or B-, Si-, and Al-C_59_ fullerenes, the energy of the independent HU drug, and the energy of the C_60_ and B-, Si-, and Al-C_59_ fullerenes, respectively.

In order to understand the degree of charge redistribution between the drug molecule and fullerene in more detail, charge density difference (CDD, Δρ) maps were calculated and defined as follows:

(2) $$\Delta \rho =\rho_{\mathrm{complex}}-\rho_{\mathrm{HU}}-\rho_{\mathrm{fullerene}\;\mathrm{or}\;\mathrm{heterofullerene}}$$

where ρ_complex_, ρ_HU_, and ρ_fullerene or heterofullerene_ are the electron density of the HU adsorbed on C_60_ or B-, Si-, and Al-C_59_ fullerenes, the electron density of the independent HU drug, and the electron density of C_60_ and B-, Si-, and Al-C_59_ fullerenes, respectively. In the CDD calculation, we put the complex into a periodic 25 × 25 × 25 Å cubic box, and the k-sampling was restricted to the Γ point. The other calculation parameters are the same as described above.

## 3. Results and Discussion

### 3.1. The Hydroxyurea Molecule Characterizations

**Figure 1:**
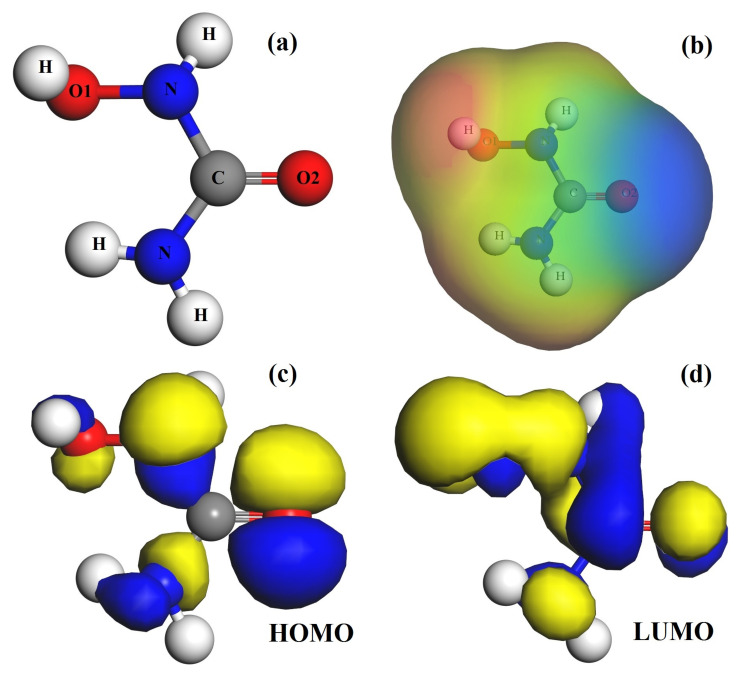
The optimized structure of hydroxyurea (HU) drug (**a**), and the molecular electrostatic potential (MEP) (**b**), HOMO (**c**) and LUMO (**d**) profiles of HU. Here, in the MEP plot the blue and red colors correspond to more negative and positive electrostatic potentials regions, respectively.

The optimized geometry, molecular electrostatic potential (MEP) plot, highest-occupied molecular orbital (HOMO) and lowest-unoccupied molecular orbital (LUMO) plots of hydroxyurea (HU) are presented in Figure 1. The MEP plot shows that the O(2) atom of HU has the highest negative electrostatic potential and, thus, could be considered as the active site when interacting with fullerene molecule [19,20,21]. Although the two N atoms also have slightly negative electrostatic potential, N atom sites probably cannot be the adsorption sites due to the steric effect [22]. Moreover, the highest density regions of HOMO and LUMO profiles are mainly localized on the O(2) atom and the H atom adjacent to O(1) atom, respectively, which is consistent with the blue and red regions on the MEP plot.

### 3.2. The Adsorption of HU on the Pristine C_60_

**Figure 2:**
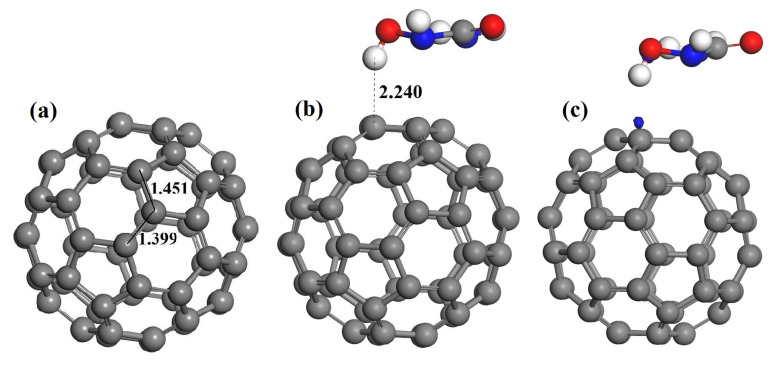
Optimized structures of C_60_ (**a**) and HU-C_60_ (**b**). All distances are in Angstrom. (**c**) The charge density difference (CDD) of HU-C_60_. The isosurface value is 0.03 a.u. In the CDD plot, the blue and yellow colors correspond to the electron density gain and loss regions, respectively.

C_60_ is composed of 20 hexagonal and 12 pentagonal rings, and C-C bonds can be classified into two types. The first one is shared between two hexagons and referred as (6-6) bond, and the second one is shared by one hexagon and one pentagon rings and named as (6-5) bond. As shown in Figure 2, the calculated bond lengths in C_60_ are 1.451 and 1.399 Å, essentially in good agreement with experimental values [44]. There are several possible adsorption configurations when HU molecule is attached to C_60_. All the high symmetry adsorption sites, including the five-membered ring, the six-membered ring, the C-C bond and C atom top, have been considered as the active sites of the C_60_. As shown in Figure 2, the most stable configuration is the H atom of HU adsorption on the top of the C atom with a distance of 2.24 Å. Upon adsorption, both the bond lengths around the adsorption C atom of C_60_ and HU molecule are nearly unchanged. In addition, the adsorption energy (Table 1) of the pristine C_60_ and HU systems is −0.271 eV. The relatively low adsorption energy and the almost unaltered geometric parameters of pristine C_60_ and the HU indicate that the interaction between them mainly occurs via noncovalent bond [45].

In order to better understand the interaction between pristine C_60_ and HU drug molecule, we further analyzed the charge transfer based on Hirshfeld charge analysis. The charge transfer from HU to pristine C_60_ is −0.047 e (Table 1), which is relatively small. Moreover, as can be seen from Table 1, the HOMO and LUMO energy levels of HU-C_60_ complex have only slightly changed compared to the initial C60, resulting in the HOMO-LUMO energy gap (Eg) also reduced very slightly, which suggests the adsorption of HU molecules has little effect on the electronic properties of C_60_ [19,20,21,22].

Figure 2c shows the charge density difference (CDD) plot of HU-C_60_ with the isosurface value of 0.03 a.u. It is apparent from this figure that few charge density difference in the region between HU and C_60_ can be observed. Besides the lower adsorption energy and charge transfer, and slightly changed energy gap, the insignificant charge density difference further confirms the weak interaction nature between HU and C_60_ [17,18]. Therefore, pristine C_60_ is not a proper drug carrier for HU. To enhance the reactivity of C_60_, we explored the effect of replacing a C atom of C_60_ with B, Si, and Al atom on the adsorption properties of HU drug.

### 3.3. The Adsorption of HU on the Doped C_60_

**Figure 3:**
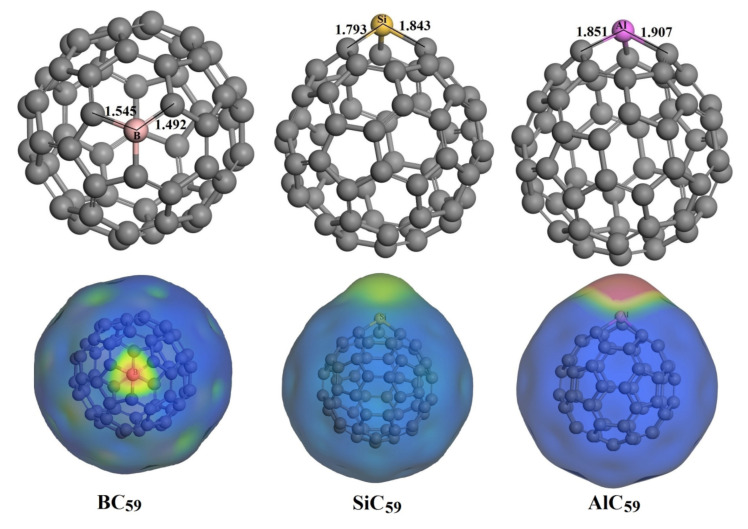
Optimized structures and the corresponding molecular electrostatic potential (MEP) plots of BC_59_, SiC_59_, and AlC_59_. Here, in the MEP plot the blue and red colors correspond to more negative and positive electrostatic potentials regions, respectively. All bond distances are in Angstrom.

The optimized structures of the doped C_60_ by B, Si, and Al atom are presented in Figure 3. Due to the larger covalent radius, all the dopant atoms caused certain deformations at the point where they were inserted in C_60_ cage. Compared with pristine fullerenes, the geometry of BC_59_ undergoes a slight deformation, and the corresponding (6-5) and (6-6) B-C bond lengths are stretched to 1.545 and 1.492 Å, respectively. In SiC_59_, the formed (6-5) and (6-6) Si-C bond lengths are 1.843 and 1.793 Å. Compared with BC_59_ and SiC_59_, the structural deformation caused by Al doping becomes more pronounced, since the Al atom is larger in size.

Meanwhile, the M-doping also significantly modifies the electronic structures of the C_60_. It can be seen from Table 1 that the HOMO-LUMO energy gap (*E*_g_) of the doped system is significantly decreased, especially for the BC_59_ and AlC_59_. In the framework of conceptual density functional theory [46], the lower *E*_g_ means higher reactivity. Therefore, doping by B, Si and Al atoms has improved the reactivity of C_60_ remarkably. In SiC_59_, the decrease of *E*_g_ is less significant than the case of BC_59_ and AlC_59_, which could be explained by the Si atom is valence isoelectronic with C atom [7].

According to the above analysis, the doping with B, Si, and Al atoms properly modify the structure and electronic properties of C_60_, which may make it possible for the delivery of HU molecule. The computed MEP plots of BC_59_, SiC_59_, and AlC_59_ are shown in Figure 3. The MEP plot, which is based on the distribution of charge density, is an efficient approach for the visualization of the reactive sites on the molecule. It is apparent from Figure 3 that the B, Si, and Al sites have the highest positive electrostatic potential in BC_59_, SiC_59_, and AlC_59_, respectively. So, the B, Si, and Al atoms act as the electrophilic sites. As mentioned above, the O(2) atom in HU molecule act as the nucleophilic sites. Therefore, we can predict that the HU molecule should absorb from its O(2) atom to the doping site of the MC_59_.

**Figure 4:**
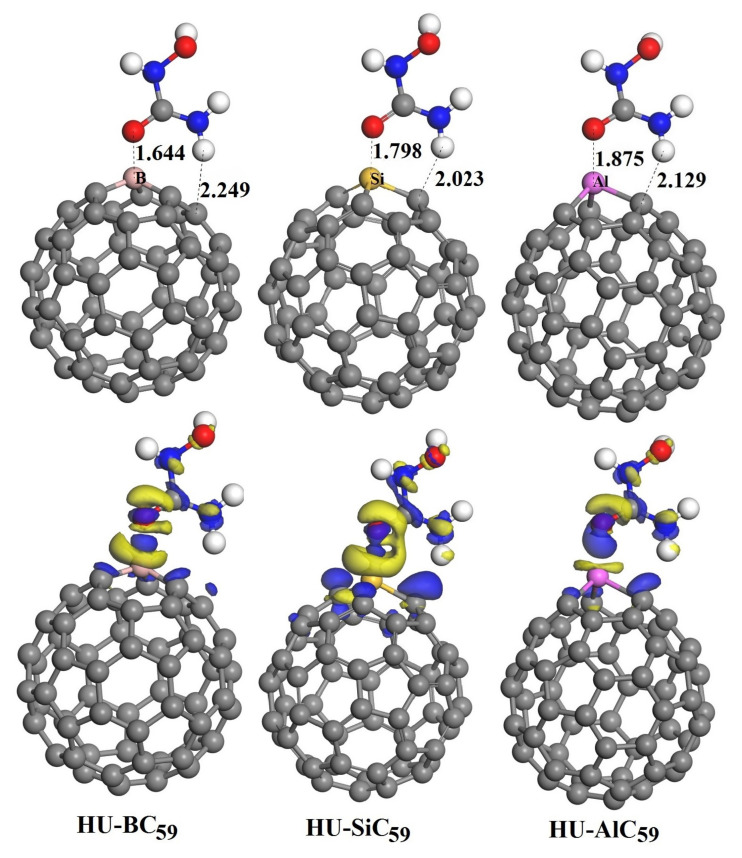
Optimized most stable structures and corresponding charge density difference (CDD) of HU-BC_59_, HU-SiC_59_, and HU-AlC_59_. All distances are in Angstrom. For comparison, the isosurface value is set to 0.03 a.u. (the same as Figure 2) for all. In the CDD plots, the blue and yellow colors correspond to the electron density gain and loss regions, respectively.

The optimized geometries of the HU-MC_59_ complexes are shown in Figure 4. The most stable structures are HU molecule interact with the B, Si, and Al atoms (electrophilic sites) of MC_59_ through its O(2) atom (nucleophilic site), which is in agreement with the MEP prediction. It can be seen from Table 1 that the adsorption energies of the HU-BC_59_, HU-SiC_59_, and HU-AlC_59_ complexes are −1.122, −1.567, and −2.174 eV, respectively. A negative value of the adsorption energy indicates that the adsorption process is exothermic and geometrically stable. Generally, the energetic threshold separating the adsorption energy of physisorption and chemisorption is about 0.5 eV per adsorbed species [47]. Due to the relatively more considerable adsorption energy, the interaction between the HU and the MC_59_ should be chemisorption [5,6,7,8]. It can be seen that the HU-AlC_59_ mixture has the largest adsorption energy and is the most stable one among the three complexes.

To further understand the interaction between HU drug molecule and MC_59_, we calculated the charge transfer based on Hirshfeld charge analysis. From Table 1, it can be seen that the amount of charge transfer of HU-MC_59_ complex (0.373, 0.429, and 0.346 *e*) is much higher than HU-C_60_ (−0.047 *e*). The CDD plots of HU-MC_59_ are also calculated and shown in Figure 4. Different to the case of HU-C_60_ (Figure 2), here we can observe obvious charge differences between the HU and MC_59_.

**Figure 5:**
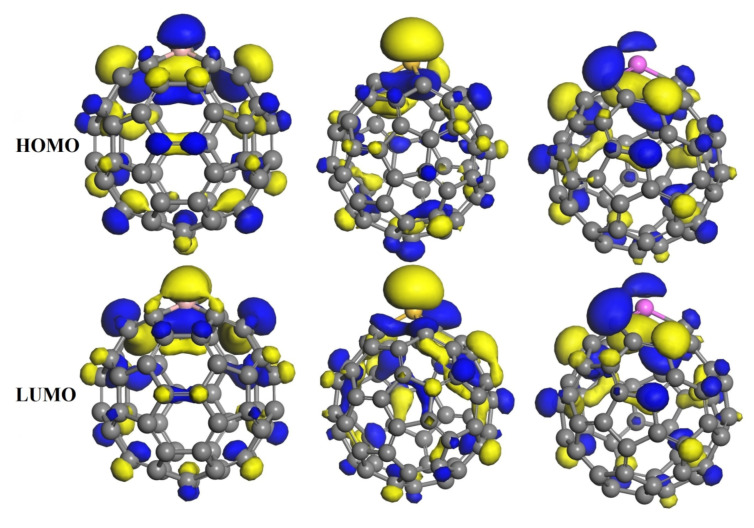
The HOMO and LUMO pictures of the BC_59_, SiC_59_, and AlC_59_ molecules.

**Figure 6:**
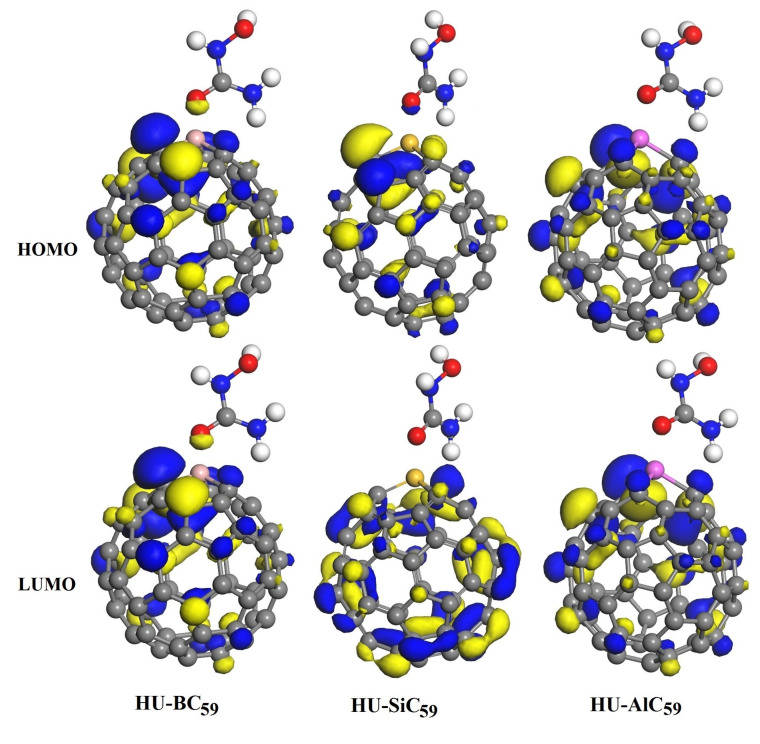
The HOMO and LUMO pictures of the most stable configurations of the HU absorbed on the BC_59_, SiC_59_, and AlC_59_ molecules.

Furthermore, as shown in Figure 5 and Figure 6, we have calculated the frontier molecular orbitals of the MC_59_ molecules and HU-MC_59_ complexes. It can be seen from Figure 5 that in MC_59_ the HOMO and LUMO are mainly distributed around the M-dopant with different orbital shapes, which is in agreement with previous reports [21,22]. However, after the HU molecule adsorbed on the MC_59_ (Figure 6), the original charge density above the M-dopant atom disappeared for both HOMO and LUMO, which indicates the relatively stronger interaction between HU and MC_59_ [7].

Overall, the moderate adsorption energy, moderate charge transfer and the obvious charge density difference confirm the chemisorption nature of HU on MC_59_. These findings suggest that heterofullerene BC_59_, SiC_59_, and AlC_59_ molecules can be used as the potential drug carrier for HU drug.

From the perspective of theoretical calculations, research strategies on the potential applications of heterofullerene in drug delivery [19,20,23] and drug detection [16,21,22] are similar. Hence, we further analyzed the possibility of MC_59_ for HU drug detection. We utilize the relation between electronic conductivity and band gap of an intrinsic semiconductor [48]:

(3) $$\sigma \propto \exp(\frac{-E_{g}}{2kT})$$

where *σ* is the electric conductivity, *E*_g_ is the band gap, *k* is known as the Boltzmann’s constant, and *T* is the thermodynamic temperature. It can be seen from Formula 3 that the electric conductivity is exponentially related to *E*_g_ at a given temperature. Thus, a smaller change in *E*_g_ results a larger variation in the electric conductivity.

Although this formula is derived for intrinsic semiconductor, it has also been widely employed to study the potential applications of nanomaterials in gas sensing [5,6,7,8,49,50] and drug detection [16,22]. Take the Al-doped ZnO nanostructure (AZO) for CO chemical sensors for example [51,52]. The experimental results show that the electronic conductivity of the AZO film increases significantly upon CO adsorption [51], which can be understood well with the reduction of the HOMO-LUMO gap of AZO molecule after the adsorption of CO [52]. The validity of Formula 3 for nanomaterials is mainly due to the relatively weak interaction between nanomaterial molecules, which makes the nanomaterial films mimic the electronic properties of the single molecule to some extent. After the HU adsorption, the HOMO-LUMO energy gap (*E*_g_) of HU-MC_59_ complexes change obviously (in the range of 13.4% to 33.7% Table 1), which would result a larger variation in the electric conductivity of MC_59_ film. Therefore, the HU molecule could be detected by MC_59_ (M = B, Si, Al) theoretically.

At the end of this article, we briefly discuss the toxicity of fullerene and its derivatives. Fullerene and its derivatives, which have been extensively studied in biomedicine, have shown excellent potential applications in many fields, such as cancer therapy and photodynamic therapy [10,11,12,13,14]. However, as stated in some review articles, fullerene and its derivatives’ presence or absence of toxicity remains a controversial issue [53,54]. Some research results show that fullerene derivatives are toxic. On the other hand, a series of tests show that fullerene derivatives are non-toxic, especially at low concentrations [53,55]. For example, the recent experimental work by Liu et al. [56] has shown that fullerene derivative Gd@C_82_(OH)_22_ can be used as a non-toxic breast cancer stem cell-specific inhibitor. Therefore, it should be pointed out that our theoretical calculation results here offer a potential carrier for HU drug, and further investigations on toxicity and clinical trials are indispensable before application.

## 4. Conclusions

In summary, with the aim to find a promising nanocarrier for HU drug delivery, we have investigated the interactions between HU drug and pristine C_60_ and heterofullerene MC_59_ (M = B, Si, Al) using density functional theory calculations in terms of adsorption geometries, adsorption energies, charge transfer, and electronic properties. It is found that the HU molecule is physisorbed on the pristine C_60_ with lower adsorption energy and negligible charge transfer. So, pristine C_60_ cannot act as the carrier for HU drug delivery. On the contrary, the HU molecule is chemisorbed on the BC_59_, SiC_59_, and AlC_59_ molecules with strong binding and can lead to finite charge transfer. Thus, it is suggested that heterofullerene BC_59_, SiC_59_, and AlC_59_ are potential candidates for HU drug delivery.

## Figures and Tables

**Table 1:** Calculated adsorption energy (*E*_ads_), HOMO energy (*E*_HOMO_), LUMO energy (*E*_LUMO_), HOMO-LUMO gap (*E*_g_), the change of *E*_g_ after the HU adsorption (∆*E*_g_), and charge transfer from the HU to fullerene (*q*_CT_) of the structures in gas phase.

Structure	*E*_ads_ (eV)	*E*_HOMO_ (eV)	*E*_LUMO_ (eV)	*E*_g_ (eV)	∆*E*_g_ (%)	*q*_CT_ (e)
C_60_	-	−5.745	−4.104	1.641	-	-
HU-C_60_	−0.271	−5.738	−4.183	1.555	−5.241	−0.047
BC_59_	-	−5.636	−5.363	0.273	-	-
HU-BC_59_	−1.122	−4.803	−4.438	0.365	33.700	0.373
SiC_59_	-	−5.806	−4.619	1.187	-	-
HU-SiC_59_	−1.567	−4.720	−3.709	1.011	−14.827	0.429
AlC_59_	-	−5.445	−5.124	0.321	-	-
HU-AlC_59_	−2.174	−4.884	−4.520	0.364	13.396	0.346
